# Changes in knee phenotype in coronal plane alignment after medial unicompartmental knee arthroplasty did not affect clinical outcomes

**DOI:** 10.1186/s42836-026-00447-x

**Published:** 2026-11-04

**Authors:** Sakolrat Lapanikorn, Aree Tanavalee, Srihatach Ngarmukos, Nonn Jaruthien, Chotetawan Tanavalee, Chavarin Amarase

**Affiliations:** 1 Biologics for Knee Osteoarthritis Research Unit, Faculty of Medicine, Chulalongkorn University, Bangkok, 10330 Thailand; 2 Department of Orthopaedics, Faculty of Medicine and King Chulalongkorn Memorial Hospital, Chulalongkorn University, Bangkok, 10330 Thailand

**Keywords:** Unicompartmental knee arthroplasty (UKA), Coronal plane alignment of the knee (CPAK), Knee phenotype, Patient-reported outcome measures (PROMs)

## Abstract

**Background:**

Studies on total knee arthroplasty (TKA) demonstrated increased positive patient-reported outcomes when the coronal plane alignment of the knee (CPAK) phenotype was preserved. However, reports on outcomes related to changes in CPAK following unicompartmental knee arthroplasty (UKA) remain limited.

**Methods:**

One hundred forty-five patients undergoing unilateral medial UKAs with pre- and postoperative full-body anteroposterior standing radiographs were enrolled in the study. Angles in the coronal plane alignment, including medial proximal tibial angle (MPTA), lateral distal femoral angle (LDFA), arithmetic hip–knee–ankle angle (aHKA), and joint line obliquity (JLO), were measured. Postoperatively, patients were grouped into restored and altered CPAK groups. With a minimum 1-year follow-up (FU), patient-reported outcome measures, including the Forgotten Joint Score-12 (FJS-12), Oxford Knee Score (OKS), and 12-Item Knee Injury and Osteoarthritis Outcome Score (KOOS-12), were evaluated and compared between the two groups.

**Results:**

Among 145 patients, 76 with true anteroposterior radiographs completed the final FU. The mean age, BMI, and FU time were 69.5 years, 27.19 kg/m2, and 23.2 months, respectively. The two most common preoperative CPAK phenotypes were type I (52.6%) and type II (32.9%). With no differences in preoperative MPTA, LDFA, Kellgren–Lawrence (KL), and Ahlbäck grade, 35 patients had their CPAK restored, and 41 had altered CPAK. The postoperative aHKA was not overcorrected toward valgus alignment (aHKA > 2 degrees) in either group, and similarly significant improvements were observed in FJS-12, OKS, and KOOS-12 scores from baseline. The altered CPAK group demonstrated significantly higher MPTA and JLO values than the restored CPAK group, corresponding to a shift in CPAK phenotype, most commonly from type I to IV or VII and from type II to VII or VIII. In addition, clinical outcomes of 65 patients with unsuitable radiographs for CPAK evaluation were similar to those of included patients in the final analysis.

**Conclusion:**

Both restored CPAK and altered CPAK patients after medial UKA showed similar improvements in short-term patient-reported outcomes at a mean 23-month FU. Mid- to long-term clinical outcomes in patients with changes in knee phenotype should be evaluated.

## Introduction

Knee osteoarthritis (OA) is a progressive, degenerative joint disease that has evolved from being viewed as simple “wear and tear” to being understood as a complex disease of the entire joint organ. It is characterized by the degradation of articular cartilage, remodeling of subchondral bone, synovial inflammation, and joint capsule fibrosis, which ultimately leads to structural failure. The progressive loss of articular cartilage, which normally absorbs shock, leads to bone-on-bone friction, causing severe pain and reduced joint space [1, 2]. Anteromedial osteoarthritis (AMOA) is a common early pattern of knee osteoarthritis. It is characterized by cartilage degeneration localized to the medial compartment of the knee, particularly in the anterior and central regions of the medial tibial plateau. In contrast, the posterior cartilage remains relatively well preserved [3]. Previous studies have reported that AMOA is present in approximately 46.9% of patients with knee osteoarthritis undergoing joint replacement surgery [4].

Recently, the concept of coronal plane alignment of the knee (CPAK) was introduced as a phenotype-based classification system to describe native knee alignment using two independent parameters derived from radiographic measurements: the arithmetic hip-knee-ankle angle (aHKA), representing constitutional limb alignment, and joint line obliquity (JLO) [5]. By combining three categories of aHKA with three categories of JLO, the CPAK system defines nine distinct knee phenotypes and provides a standardized framework for evaluating coronal knee alignment in both native and post-arthroplasty knees [5]. Several studies have investigated the relationship between CPAK phenotypes and clinical outcomes following total knee arthroplasty (TKA), showing that preserving the native knee phenotype through kinematic alignment improves functional outcomes and increases patient satisfaction [6–8]. Conversely, some studies have demonstrated that postoperative changes in CPAK phenotype were associated with worse or no significant differences in patient-reported outcomes between preserved and altered CPAK phenotypes following TKA [9–13]. These conflicting findings indicate that the relationship between coronal alignment phenotype and clinical outcomes remains controversial.

Compared with TKA, unicompartmental knee arthroplasty (UKA) is generally considered a joint-resurfacing procedure, and postoperative coronal alignment is expected to remain close to the patient’s native alignment. Although previous studies have shown that coronal alignment and joint-line orientation often change following UKA, potentially altering the CPAK phenotype [14–16]. Nevertheless, whether preserving the native coronal alignment phenotype translates into better patient-reported outcomes remains contested, with conflicting results reported across studies [14, 17–19].

The purpose of this study was to evaluate clinical outcomes, including the Forgotten Joint Score-12 (FJS-12), Oxford Knee Score (OKS), and the 12-item Knee Injury and Osteoarthritis Outcome Score (KOOS-12), of medial UKA patients related to changes in CPAK phenotype.

## Materials and methods

This retrospective cohort study was approved by the Institutional Review Board of the Faculty of Medicine, Chulalongkorn University, Bangkok, Thailand (IRB No. 0779/68). The study protocol was registered at Thai Clinical Trials (https://thaiclinicaltrials.org) under the identification number TCTR20260318001, with a registration date of 18 March 2026. All study procedures were conducted in accordance with relevant guidelines and regulations.

Between September 2022 and April 2024, 145 patients aged > 50 years with anteromedial osteoarthritis (AMOA) who underwent a unilateral unicompartmental knee arthroplasty (UKA) with a minimum follow-up (FU) of 1 year were enrolled. Preoperatively, a full-body anteroposterior (AP) radiograph was obtained with the patient standing on full weight-bearing. The lower extremities were positioned with each patella facing forward and the knee fully extended, as described by Paley and Pfeil [20]; the feet were flat on the ground, barefoot, with toes pointing forward and no rotation from the midline; patients were allowed to place their hands on handlebars for balance; the technicians additionally advised patients to distribute their weight evenly between both legs. The entire cervical to sacral spine, pelvis, both hips, knees, and ankles were included; all vertebrae were in a neutral rotation position; bilateral shoulder girdles (including the clavicle, scapula, and proximal humerus) were symmetrical; pelvis was in a neutral position; and both lower extremities were oriented with each patella facing forward and each knee maximally extended. The angle in the coronal plane, including medial proximal tibial angle (MPTA), lateral distal femoral angle (LDFA), aHKA, and JLO, was measured according to the method described by MacDessi et al. [5]. All knees were classified into nine phenotypes according to the CPAK classification, defined as preoperative phenotypes. The exclusion criteria included patients with unsuitable radiographs, including different foot positions during taking radiographs between the pre- and postoperative periods; non-true AP radiographic images; component rotation affecting angle measurements; tibial component tilting in the sagittal plane affecting angle measurements; use of a gait aid; and inability to complete questionnaires.

All surgeries were performed using two medial unicompartmental knee systems: the Oxford Partial Knee (Zimmer Biomet, Warsaw, IN, USA) and the Journey II Unicompartmental Knee System (Smith & Nephew, Memphis, TN, USA), through a minimally invasive mini-midvastus approach and using manual instruments, by three experienced arthroplasty surgeons (AT, SN and CT). The surgical alignment philosophy was minimal tibial bone cut and targeted restoration of native pre-arthritic limb alignment, avoiding overcorrection of the neutral mechanical axis, without a fixed numerical alignment target and without intraoperative soft-tissue release.

**Fig. 1:**
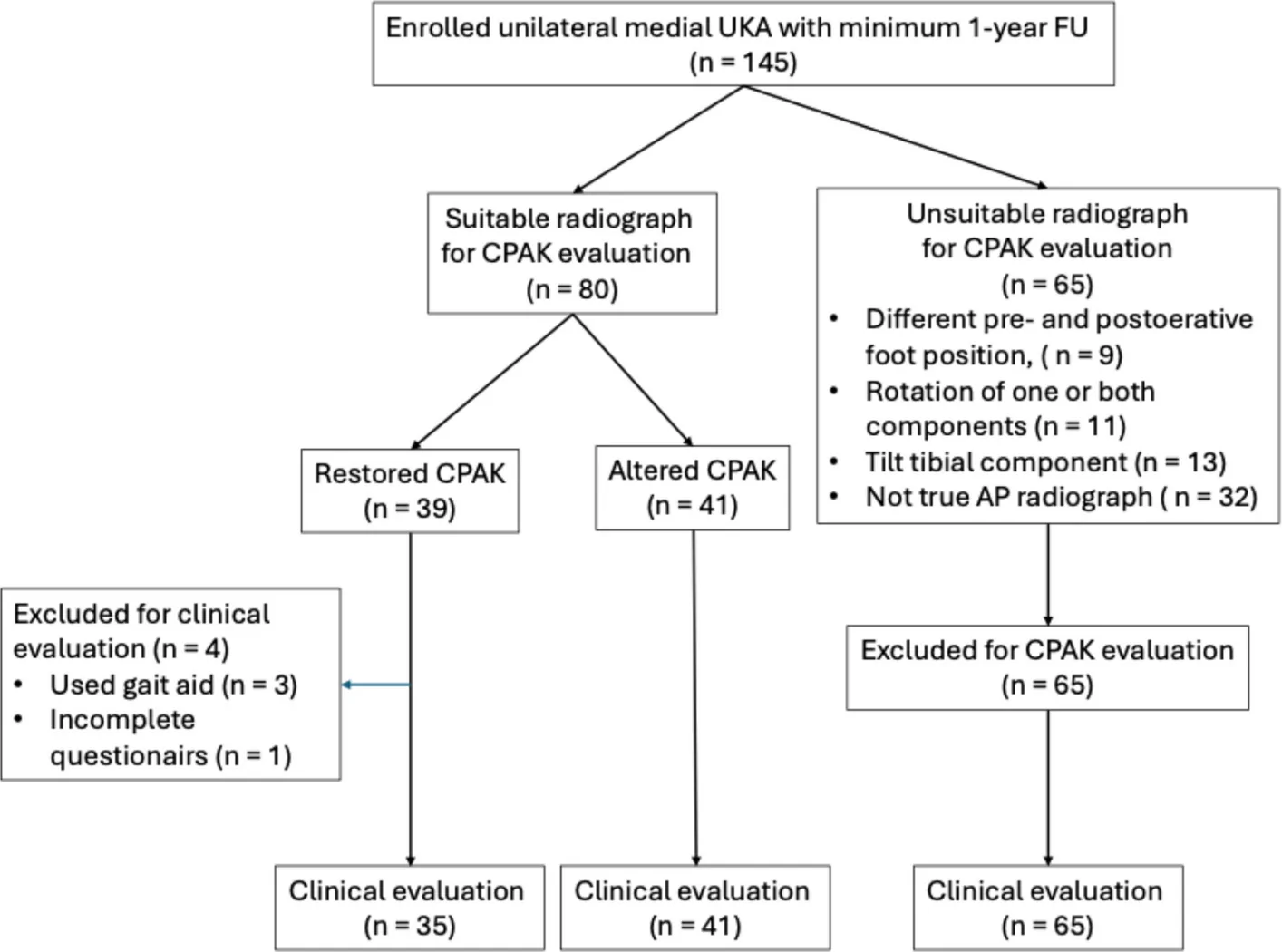
Flow diagram of the study group. UKA: unicompartmental knee arthroplasty; CPAK: coronal plane alignment of the knee

All patients were evaluated preoperatively using patient-reported outcome measures (PROMs) based on questionnaire responses, including the FJS-12, OKS, and KOOS-12. At a minimum of 1 year after surgery, all patients underwent the same PROM evaluations, and patient satisfaction was assessed on a 0- to 10-point scale, with knee range of motion measured. Using the same radiographic protocol, postoperative full-body AP radiographs were obtained in all patients; similar angle measurements were taken, and patients were classified according to the CPAK phenotype, and the resulting postoperative CPAK phenotypes were defined. Patients were then divided into two groups: the restored CPAK group, in which the postoperative CPAK phenotype remained unchanged from those measured in the preoperative period, and the altered CPAK group, in which the postoperative CPAK phenotype differed from the preoperative classification. The flow diagram of the study is shown in Fig. 1.

The primary outcome was to compare FJS-12 scores at the latest FU between patients in the restored CPAK group and the altered CPAK group. Secondary outcomes included comparing OKS, KOOS-12, patient satisfaction, and knee range of motion between the two groups.

### Landmarks and radiographic measurements for LDFA, MPTA, JLO, and aHKA

**Fig. 2:**
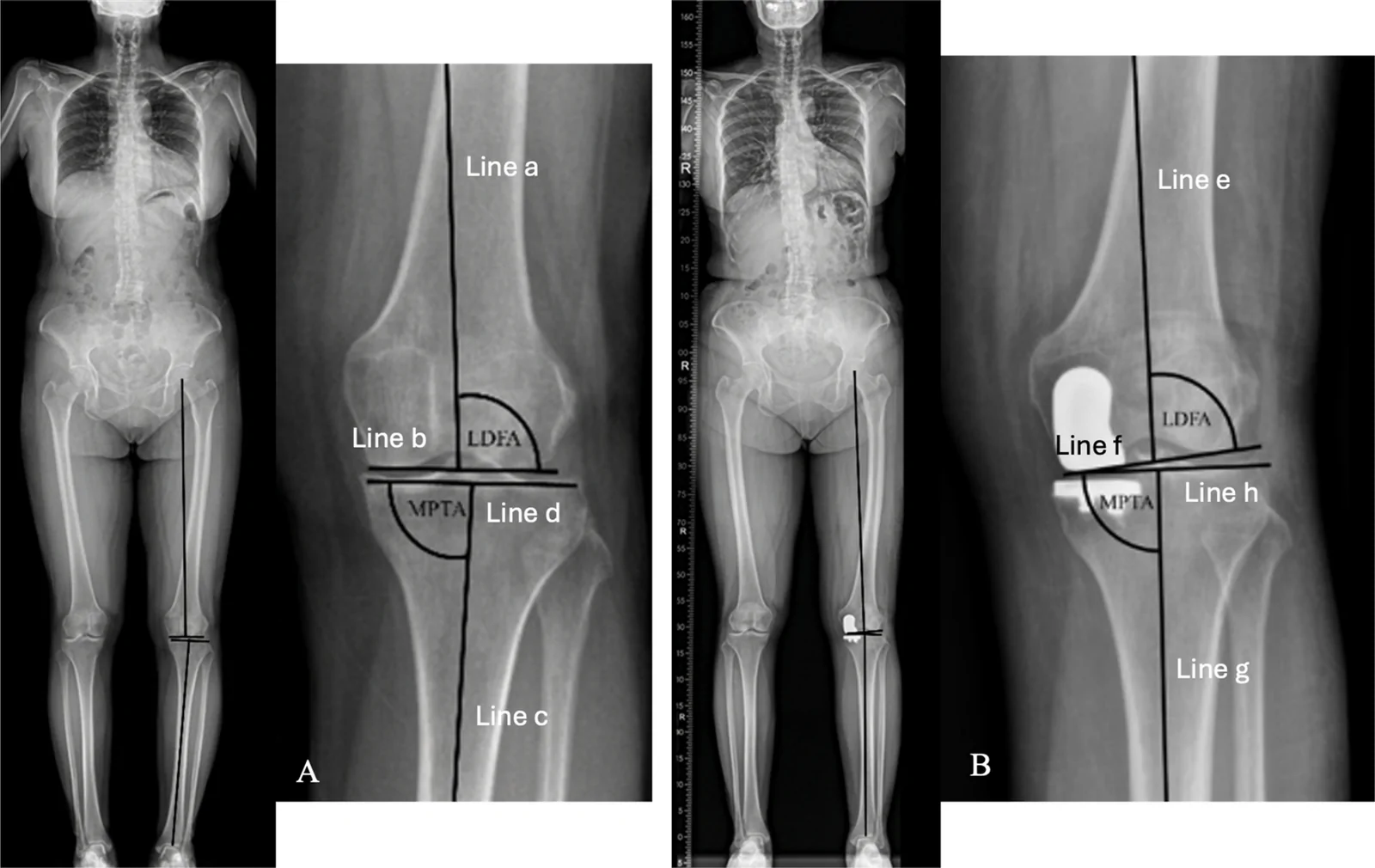
Full-body anteroposterior standing radiographs illustrating reference lines for measurements of the medial proximal tibial angle (MPTA) and lateral distal femoral angle (LDFA). **A** Preoperative radiographs: a: A line drawn from the centers of the hip to the distal femur; b: A line connecting the lowest points of both femoral condyles; c: A line drawn from the centers of the proximal tibia to the ankle; d: A line connecting the highest parts of both tibial plateaus. **B** Postoperative radiographs: e: A line drawn from the centers of the hip to the distal femur; f: A line connecting the lowest points of the lateral femoral condyle and the femoral component; g: A line drawn from the centers of the proximal tibia to the ankle; h: A line connecting the highest part of the lateral tibial plateau and the contact point of the femoral and polyethylene of the tibial component

All radiographic measurements were performed by two independent evaluators (SL and NJ). Preoperatively, according to MacDessi et al. [5], with a full-body AP standing radiograph, A line drawn from the center of the hip to the center of the distal femur was made (Line a), and a line connecting the lowest points of both femoral condyles was drawn (Line b). The angle formed by Lines a and b, measured toward the lateral femoral condyle, was defined as the preoperative LDFA. On the tibial side, a line drawn from the tibial center to the center of the ankle was made (Line c), and a line connecting the highest parts of both tibial plateaus was drawn (Line d). The angle formed by Lines c and d, measured toward the medial tibial plateau, was defined as the preoperative MPTA. The preoperative aHKA was the angle formed by preoperative MPTA − LDFA. The preoperative JLO was the angle formed by preoperative MPTA + LDFA (Fig. 2A).

Postoperatively, based on MacDessi et al.’s [5] original method, with a full-body AP standing radiograph, a line drawn from the center of the hip to the center of the distal femur was made (Line e). A line connecting the lowest points of the lateral femoral condyle and the femoral component was drawn (Line f). The angle formed by Lines e and f, measured toward the lateral femoral condyle, was defined as the postoperative LDFA. On the tibial side, a line drawn from the tibial center to the center of the ankle was made (Line g), and a line connecting the highest parts of the lateral tibial plateau and the contact point of the femoral and polyethylene of the tibial component was drawn (Line h). The angle formed by Lines g and h, measured toward the medial tibial plateau, was defined as the postoperative MPTA. The postoperative aHKA was the angle formed by postoperative MPTA − LDFA. The postoperative JLO was the angle formed by postoperative MPTA + LDFA (Fig. 2B).

### Statistical analysis

Statistical analysis was performed using SPSS version 30 (IBM Corp., Armonk, NY, USA). The sample size was calculated according to the FJS-12 as the primary outcome, based on the UKA study by Longo et al. [21], using a minimal clinically important difference (MCID) of 12.5 points on the FJS-12 and a standard deviation (SD) of 11.4. An alpha level of 0.05 and a statistical power of 90% (1 − β = 0.90) were used; the calculated sample size was at least 38 patients. The validated Patient Acceptable Symptom State (PASS) threshold for FJS-12 (72.9 points) [20] was used to classify patients as achieving a satisfactory outcome (see Results). Continuous variables were expressed as mean ± SD and compared between the restored and altered CPAK groups using the independent t-test or Mann–Whitney U test, as appropriate. A one-way ANOVA was used to compare the restored CPAK, altered CPAK, and unsuitable-radiographs groups for clinical outcome measures. Categorical variables were presented as counts and percentages and analyzed using the chi-square test or Fisher’s exact test. A *p*-value < 0.05 was considered statistically significant. Regarding radiographic measurements, the intra-observer and inter-observer intraclass correlation coefficients (ICCs) were expected to be > 0.75, and the agreement for CPAK phenotype classification was expected to be Kappa > 0.8.

## Results

Sixty-five patients were excluded from radiographic CPAK evaluation because of unsuitable pre- and postoperative AP radiographs, for which the intra- and inter-observer ICCs were less than 0.5 for any of the measured angles, resulting in poor intra- and inter-observer Kappa agreement for CPAK phenotypes (Kappa < 0.5). Among the 80 patients with suitable radiographs, four patients who demonstrated a restored CPAK phenotype were excluded from the final analytic cohort: three because of gait-aid use, which could affect weight-bearing radiographs, and one because of incomplete questionnaires. Therefore, 76 patients with suitable AP radiographs were included in the final analysis regarding CPAK phenotype, with intra-observer ICCs of 0.93 for MPTA and 0.87 for LDFA, and inter-observer ICCs of 0.90 for MPTA and 0.84 for LDFA. Agreement for CPAK phenotype classification was substantial to almost perfect (Kappa = 0.82 intra-observer; Kappa = 0.78 inter-observer). For the clinical outcome comparison, these 76 patients were analyzed together with the 65 patients who had unsuitable radiographs, resulting in a clinical evaluation cohort of 141 patients. The mean age of the studied group was 69.5 years, and the mean body mass index (BMI) was 27.19 kg/m^2^, with a female predominance. The demographic data of the studied group are shown in Table 1, with no significant differences in baseline characteristics between the restored and altered CPAK groups, including age, sex, BMI, side, FU duration, and knee OA grading according to Kellgren–Lawrence and Ahlbäck.

**Table 1:** Baseline demographic and radiographic characteristics of patients included in the CPAK analysis

**Variable**	**Restored CPAK** **(*n* = 35)**	**Altered CPAK** **(*n* = 41)**	***p*-value**
Age (years)	68.80 ± 6.91	70.12 ± 6.58	0.398
Sex (female)	85.7% (30/35)	85.4% (35/41)	0.966
BMI (kg/m^2^)	27.29 ± 3.92	27.07 ± 3.49	0.804
Side (right)	54.3% (19/35)	51.2% (21/41)	0.971
Follow-up (months)	22.17 ± 9.58	23.98 ± 8.37	0.384
**Kellgren-Lawrence grading**			0.606
Grade II	42.9% (15/35)	48.8% (20/41)	
Grade III	57.1% (20/35)	51.2% (21/41)	
**Ahlbäck grading**			0.987
Grade I	2.9% (1/35)	2.4% (1/41)	
Grade II	94.3% (33/35)	95.1% (39/41)	
Grade III	2.9% (1/35)	2.4% (1/41)	
**Radiographic parameters**			
MPTA (°)	84.77 ± 2.50	84.83 ± 2.87	0.925
LDFA (°)	90.83 ± 3.62	91.20 ± 3.86	0.671
aHKA (°)	− 6.06 ± 5.05	− 6.37 ± 5.14	0.793
JLO (°)	175.60 ± 3.62	176.02 ± 4.46	0.649
**Preoperative CPAK Phenotype**	**Restored CPAK group**	**Altered CPAK group**	**Total (*n* = 76)**
Type I	18 (51.4%)	22 (53.7%)	40 (52.6%)
Type II	13 (37.1%)	12 (29.3%)	25 (32.9%)
Type III	1 (2.9%)	0 (0%)	1 (1.3%)
Type IV	0 (0%)	3 (7.3%)	3 (3.9%)
Type V	0 (0%)	3 (7.3%)	3 (3.9%)
Type VI	0 (0%)	0 (0%)	0 (0.0%)
Type VII	3 (8.6%)	1 (2.4%)	4 (5.2%)
Type VIII	0 (0%)	0 (0%)	0 (0.0%)
Type IX	0 (0%)	0 (0%)	0 (0.0%)

*CPAK* Coronal plane alignment of the knee, *MPTA* Medial proximal tibial angle, *LDFA* Lateral distal femoral angle, *aHKA* Arithmetic Hip Knee Ankle angle, *JLO* Joint line obliquity

**Fig. 3:**
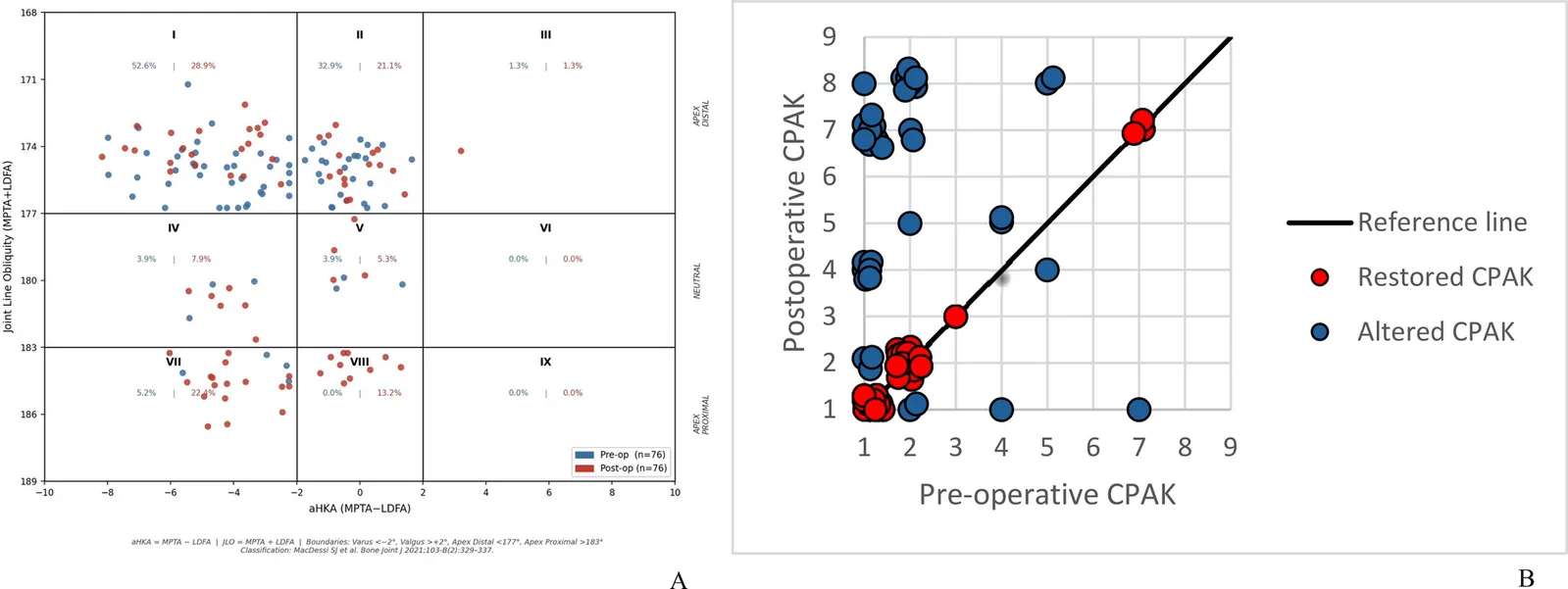
Changes in CPAK phenotypes from preoperative to postoperative assessment among patients included in the CPAK analysis. **A** Distribution of CPAK phenotypes I–IX preoperatively (blue) and postoperatively (red). Types I and II were the most common preoperative phenotypes, whereas Types I and VII were the most common postoperatively. **B** Preoperative-to-postoperative CPAK phenotype transitions. Patients in the restored CPAK group are shown in red and those in the altered CPAK group in blue. Among patients with altered CPAK phenotypes, Type I most commonly changed to Types VII and IV, whereas Type II most commonly changed to Types VIII and VII

The two most common preoperative CPAK phenotypes were type I (52.6%) and type II (32.9%), and postoperative CPAK phenotypes were type I (28.9%) and type VII (22.4%), respectively, as shown in Fig. 3A. Thirty-five patients (46%) retained their original CPAK phenotype postoperatively (restored CPAK group), while 41 (54%) shifted to a different phenotype (altered CPAK group) (Fig. 3B). There was no significant difference in postoperative aHKA between the restored and the altered groups (− 3.11 ± 2.21° vs. − 2.27 ± 1.88°; *p* = 0.079); however, the restored CPAK group showed significantly lower postoperative MPTA (87.63 ± 1.40° vs. 88.80 ± 2.23°; *p* = 0.007) and JLO (178.37 ± 2.65° vs. 179.88 ± 3.55°; *p* = 0.038). The mean MPTA change (ΔMPTA) was significantly lower in the restored group (2.86 ± 1.70° vs. 3.98 ± 2.58°; *p* = 0.027), while the mean LDFA did not differ significantly between groups (90.74 ± 1.36° vs. 91.07 ± 1.77°; *p* = 0.360), as shown in Table 2.

**Table 2:** Comparing changes in radiographic parameters between restored and altered CPAK groups. Postoperative MPTA and JLO differed significantly between the two groups, whereas there was no difference in postoperative aHKA

**Variable**	**Restored CPAK (*n* = 35)**	**Altered CPAK (*n* = 41)**	***p*-value**
Postoperative MPTA (°)	87.63 ± 1.40	88.80 ± 2.23	0.007*
Postoperative LDFA (°)	90.74 ± 1.36	91.07 ± 1.77	0.360
Postoperative aHKA (°)	− 3.11 ± 2.21	− 2.27 ± 1.88	0.079
Postoperative JLO (°)	178.37 ± 2.65	179.88 ± 3.55	0.038*
**Alignment Change**			
ΔMPTA (°)	2.86 ± 1.70	3.98 ± 2.58	0.027*
ΔLDFA (°)	− 0.09 ± 3.10	− 0.12 ± 3.58	0.962
ΔaHKA (°)	2.94 ± 3.61	4.10 ± 4.61	0.225
ΔJLO (°)	2.77 ± 3.46	3.85 ± 4.20	0.223
**Postoperative CPAK Phenotype**	Restored CPAK group*	**Altered CPAK group**	**Total (*n* = 76)**
Type I	18 (51.4%)	4 (9.8%)	22 (28.9%)
Type II	13 (37.1%)	3 (7.3%)	16 (21.1%)
Type III	1 (2.9%)	0 (0.0%)	1 (1.3%)
Type IV	0 (0.0%)	6 (14.6%)	6 (7.9%)
Type V	0 (0.0%)	4 (9.8%)	4 (5.3%)
Type VI	0 (0.0%)	0 (0.0%)	0 (0.0%)
Type VII	3 (8.6%)	14 (34.1%)	17 (22.4%)
Type VIII	0 (0.0%)	10 (24.4%)	10 (13.2%)
Type IX	0 (0.0%)	0 (0.0%)	0 (0.0%)

*CPAK* Coronal plane alignment of the knee, *MPTA* Medial proximal tibial angle, *LDFA* Lateral distal femoral angle, *aHKA* Arithmetic hip-knee-ankle angle, *JLO* Joint line obliquity

^*^The restored CPAK group’s postoperative phenotype distribution is identical to its preoperative distribution as shown in Table 1

Despite this difference in postoperative MPTA and JLO between groups, at a mean FU of 23.2 months, functional outcomes were favorable in both restored and altered CPAK groups, with no significant difference in FJS-12 (85.34 ± 8.12 vs. 86.93 ± 6.99; *p* = 0.370), OKS (41.06 ± 4.52 vs. 40.73 ± 4.78; *p* = 0.761), KOOS-12 (86.24 ± 11.51 vs. 85.66 ± 11.76; *p* = 0.829), patient satisfaction (8.94 ± 0.97 vs. 8.93 ± 1.03; *p* = 0.945), or maximum knee flexion (124.29 ± 5.58° vs. 124.88 ± 5.53°; *p* = 0.645), as shown in Table 3. According to the validated PASS threshold for FJS-12, most patients in both groups achieved a patient-acceptable symptom state, with no significant difference between the two groups (*p* = 0.461). With a similar mean FU, there were no significant differences among the restored CPAK, altered CPAK, and unsuitable-radiographs groups in FJS-12 (85.34 ± 8.12 vs. 86.93 ± 6.99 vs. 85.38 ± 6.19; *p* = 0.483), OKS (41.06 ± 4.52 vs. 40.73 ± 4.78 vs. 39.52 ± 3.63; *p* = 0.157), KOOS-12 (86.24 ± 11.51 vs. 85.66 ± 11.76 vs. 85.31 ± 7.66; *p* = 0.907), patient satisfaction (8.94 ± 0.97 vs. 8.93 ± 1.03 vs. 8.75 ± 0.85; *p* = 0.521), or maximum knee flexion (124.29 ± 5.58° vs. 124.88 ± 5.53° vs. 123.92 ± 5.04°; *p* = 0.668), as shown in Table 3.

**Table 3:** Comparison of patient-reported outcome measures, satisfaction, and maximum knee flexion among the restored CPAK, altered CPAK, and unsuitable-radiograph groups at the latest follow-up. No significant differences were observed among the three groups

Variable	Restored CPAK (*n* = 35)	Altered CPAK (*n* = 41)	Unsuitable radiographs (*n* = 65)	*p*-value^2^
FJS-12	85.34 ± 8.12	86.93 ± 6.99	85.38 ± 6.19	0.483
	*p*-value^1^ = 0.370			
OKS	41.06 ± 4.52	40.73 ± 4.78	39.52 ± 3.63	0.157
	*p*-value^1^ = 0.761			
KOOS-12	86.24 ± 11.51	85.66 ± 11.76	85.31 ± 7.66	0.907
	*p*-value^1^ = 0.829			
Satisfaction	8.94 ± 0.97	8.93 ± 1.03	8.75 ± 0.85	0.521
	*p*-value^1^ = 0.945			
Max flexion (°)	124.29 ± 5.58	124.88 ± 5.53	123.92 ± 5.04	0.668
	*p*-value^1^ = 0.645			

*FJS-12* Forgotten Joint Score-12, *OKS* Oxford Knee Score, *KOOS-12* 12-item Knee Injury and Osteoarthritis Outcome Score, *CPAK* Coronal plane alignment of the knee

*p*-value^1^: Independent t-test comparing restored vs. altered CPAK groups

*p*-value^2^: One-way ANOVA comparing restored, altered, and unsuitable-radiographs groups

## Discussion

The present study found that, at a mean FU of 23.2 months following UKA, among 76 patients with true AP radiographs, 54% of patients who exhibited a change in CPAK phenotype had clinical outcomes similar to those in the restored CPAK group, as measured by FJS-12, OKS, and KOOS-12 scores. With similar postoperative aHKA, the altered CPAK group had significantly higher postoperative MPTA and JLO values.

In the present study, there was low agreement in intra- and interobserver ICCs (< 0.5) in all measured angles in 65 excluded patients due to unsuitable AP radiographs, consistent with previous reports on variations in radiographic findings affecting angle measurement accuracy [21, 22]. Considering postoperative angle measurements after TKA based on the method described by MacDessi et al. [5], landmarks for the horizontal reference line measuring the LDFA and MPTA; the contact points of the medial and lateral femoral and tibial components are clearly applied by all investigators. However, radiographic measurements of LDFA and MPTA after UKA varied mainly with the landmark in the lateral compartment of the knee used to draw the reference line. A recent systematic review reported high variability in MPTA measurements, with different landmarks leading to different tilts of the proximal tibia’s reference line, resulting in up to a 3° change in MPTA and potentially a change in CPAK phenotype [22]. Most studies used the midpoint of the joint space in the lateral compartment as the landmark to draw a line to the lowest point of the femoral component, which served as the reference line for MPTA and LDFA [18, 23–25]; however, in agreement with the study of Vossen et al. [17], we used the line from the lateral native tibial plateau, which is lower than the former landmark, to the lowest point of the femoral component as the reference line for the MPTA and the line from the lowest point of the lateral femoral condyle to the lowest point of the femoral component as the reference line for the LDFA, which we believed they corresponded to the method used at the preoperative measurements. By using the same landmark at the medial side of the knee and different landmarks on the lateral side to measure MPTA and LDFA, one may expect that lowering the landmark for the tibia in the lateral compartment would increase the MPTA, whereas elevating the landmark for the femur in the lateral compartment would decrease the LDFA. Using a different measurement method could explain, in part, the high rate of altered CPAK observed in the present study (54%), since a landmark-dependent difference of up to 3° in MPTA as reported in the systematic review above is sufficient to shift a knee across a CPAK phenotype boundary. This suggests that some proportion of the observed “change” reflects measurement-method sensitivity rather than true postoperative phenotype migration, and the 54% figure should be interpreted with this in mind. Several studies reported preserved preoperative knee phenotypes in most knees [8, 18, 26]. Although the magnitude of aHKA change did not differ significantly between groups, the altered CPAK group had to compensate with higher mean MPTA values and increasing JLO values.

Preoperatively, the two most common knee phenotypes in the present study were types I and II, consistent with previous reports [18, 19, 27]. The percentage of type I in our study (52.6%) and in Zhao and colleagues’ study [25] (54%), which included Asian patients, was much higher than that reported in Western patients (35–46%), suggesting that low MPTA is common among Asians. Although the present study demonstrated a decreased percentage of type I (from 52.6% to 28.9%) after medial UKA, type I remained the most common postoperative knee phenotype, consistent with a recent study in a series of over 300 patients [19].

For medial UKA, in patients with low MPTA, minimal tibial bone cut is recommended to avoid the risk of medial tibial fracture [15, 26]. While the minimum thickness of the tibial component, including polyethylene, in most UKA systems is 8 mm, a minimal tibial bone cut in low MPTA tibiae tends to increase postoperative MPTA and JLO. Therefore, with the concept of a minimal tibial bone cut and avoidance of overcorrection of the aHKA, there is a tendency for CPAK to change from type I to IV or VII, and from type II to VII or VIII, which may be a reasonable explanation for patients with altered CPAK after surgery. In addition, the target postoperative aHKA after medial UKA usually ranges from a negative value to 0 degrees; therefore, a postoperative aHKA of − 2 degrees is not uncommon. While the boundary of CPAK phenotype I versus II, IV versus V, and VII versus VIII is determined by the aHKA of − 2 degrees, variation in CPAK determination may occur in those patients with postoperative aHKA of − 2 degrees.

Several studies reported that preserving the same knee phenotype in medial UKA yielded better clinical outcomes [15–18, 25], while a limited number of studies reported no differences. In a recent study by Sahbat Y et al. with a mean FU of 66 months, they found that over 60% of medial UKAs had CPAK phenotype changes with a decrease in types I and IV in the postoperative distribution and an increase in all other types, while all clinical outcome parameters were similar between altered and restored CPAK groups [19]. Consistent with the study by Sahbat Y et al., the clinical outcomes in the altered CPAK group in the present study were similar to those in the restored CPAK group.

Unlike TKA, which involves complete resurfacing of the joint and substantial alteration of soft-tissue balance, UKA preserves both cruciate ligaments, the native meniscus in the unaffected compartments, and much of the proprioceptive and kinematic function of the knee, which is defined as a resurfacing procedure of the knee joint without ligament release.

Postoperatively, the ligament tension is constrained by the retained native soft-tissue envelope; therefore, the functional consequence in coronal alignment is less after UKA than after TKA, in which the ligaments are more extensively released and rebalanced. This aligns with how UKA works: it aims to keep the joint line close to its natural position rather than forcing it to a fixed alignment target, and joint-line position is known to directly affect limb alignment after surgery [24]. Recent studies have demonstrated that postoperative HKA after medial UKA closely reflects preoperative aHKA, suggesting that constitutional coronal alignment may be largely preserved after surgery [28, 29]. In our cohort, we observed numerical postoperative changes in aHKA, as well as in MPTA and JLO. However, the magnitude of aHKA change did not differ significantly between the restored and altered CPAK groups, although both groups demonstrated numerical changes from their preoperative values. This difference may partly reflect variations in surgical technique and alignment targets. In our series, we used a minimal tibial bone-resection strategy to avoid the risk of medial tibial fracture, combined with the no-overcorrection concept; the postoperative coronal alignment parameters changed while maintaining overall varus alignment. Although differences in surgical concepts and procedures for medial UKA affected the shift in CPAK, results still supported positive clinical outcomes. Further studies on more detailed aspects, such as gait analysis and a longer FU, may help identify the differences.

This study has several limitations. First, its retrospective observational design carried an inherent risk of selection and information bias. Nevertheless, the distribution of preoperative CPAK phenotype was similar to that reported in several previous studies [15–17]. Second, a substantial number of cases were excluded from the final CPAK analysis; however, most were due to improper radiographs, reflecting poor intra- and interobserver ICC agreement across all measured angles, while the functional outcomes of excluded patients were not different from those of included patients for CPAK-related analyses. Third, the FU duration was limited to approximately 2 years. This was due to the availability of the full-body standing radiograph from mid 2022, which precluded prediction of long-term clinical outcomes or further radiographic changes in limb alignment and component fixation related to restored or altered knee phenotypes. Fourth, this was a single-center study in a Thai population, limiting generalizability to other ethnic groups. Fifth, we did not perform biomechanical or gait analysis to assess joint loading and kinematics. Sixth, longer follow-up is needed to evaluate implant survivorship and progression of lateral compartment OA. Lastly, a coronal spinal shift detected on a full-body AP radiograph indicated whether spinal imbalance in some patients might affect postoperative lower-limb alignment or clinical outcomes. Further studies regarding clinical outcomes in patients with coronal spinal imbalance are needed.

In conclusion, the medial UKA could alter the preoperative CPAK distribution while short-term clinical outcomes between those with restored and altered CPAK phenotypes were similar. Further studies with longer follow-up are needed to determine the impact of these alignment changes on implant survivorship and disease progression.

## Data Availability

No datasets were generated or analysed during the current study.
